# Physician Specialty Differences in Unprofessional Behaviors Observed and Reported by Coworkers

**DOI:** 10.1001/jamanetworkopen.2024.15331

**Published:** 2024-06-06

**Authors:** William O. Cooper, Gerald B. Hickson, Roger R. Dmochowski, Henry J. Domenico, Frederick E. Barr, Cynthia L. Emory, Jill Gilbert, Gary E. Hartman, Marie M. Lozon, William Martinez, Janesta Noland, Steven A. Webber

**Affiliations:** 1 Departments of Pediatrics and Health Policy, Center for Patient and Professional Advocacy, Vanderbilt University Medical Center, Nashville, Tennessee; 2 Department of Pediatrics, Center for Patient and Professional Advocacy, Vanderbilt University Medical Center, Nashville, Tennessee; 3 Department of Urologic Surgery, Center for Patient and Professional Advocacy, Vanderbilt University Medical Center, Nashville, Tennessee; 4 Department of Biostatistics, Vanderbilt University Medical Center, Nashville, Tennessee; 5 Arkansas Children’s Hospital, Little Rock; 6 Department of Orthopaedic Surgery, Wake Forest University School of Medicine, Winston-Salem, North Carolina; 7 Department of Medicine, Vanderbilt University Medical Center, Nashville, Tennessee; 8 Department of Surgery, Stanford University School of Medicine, Palo Alto, California; 9 Departments of Emergency Medicine and Pediatrics, University of Michigan Medical School, Ann Arbor; 10 Department of Medicine, Center for Patient and Professional Advocacy, Vanderbilt University Medical Center, Nashville, Tennessee; 11 Department of Pediatrics, Stanford University School of Medicine, California and Stanford Medicine Children’s Health, Palo Alto, California; 12 Department of Pediatrics, Vanderbilt University Medical Center, Nashville, Tennessee

## Abstract

**Importance:**

Because unprofessional behaviors are associated with patient complications, malpractice claims, and well-being concerns, monitoring concerns requiring investigation and individuals identified in multiple reports may provide important opportunities for health care leaders to support all team members.

**Objective:**

To examine the distribution of physicians by specialty who demonstrate unprofessional behaviors measured through safety reports submitted by coworkers.

**Design, Setting, and Participants:**

This retrospective cohort study was conducted among physicians who practiced at the 193 hospitals in the Coworker Concern Observation Reporting System (CORS), administered by the Vanderbilt Center for Patient and Professional Advocacy. Data were collected from January 2018 to December 2022.

**Exposure:**

Submitted reports concerning communication, professional responsibility, medical care, and professional integrity.

**Main Outcomes and Measures:**

Physicians’ total number and categories of CORS reports. The proportion of physicians in each specialty (nonsurgeon nonproceduralists, emergency medicine physicians, nonsurgeon proceduralists, and surgeons) who received at least 1 report and who qualified for intervention were calculated; logistic regression was used to calculate the odds of any CORS report.

**Results:**

The cohort included 35 120 physicians: 18 288 (52.1%) nonsurgeon nonproceduralists, 1876 (5.3%) emergency medicine physicians, 6743 (19.2%) nonsurgeon proceduralists, and 8213 (23.4%) surgeons. There were 3179 physicians (9.1%) with at least 1 CORS report. Nonsurgeon nonproceduralists had the lowest percentage of physicians with at least 1 report (1032 [5.6%]), followed by emergency medicine (204 [10.9%]), nonsurgeon proceduralists (809 [12.0%]), and surgeons (1134 [13.8%]). Nonsurgeon nonproceduralists were less likely to be named in a CORS report than other specialties (5.6% vs 12.8% for other specialties combined; difference in percentages, −7.1 percentage points; 95% CI, −7.7 to −6.5 percentage points; *P* < .001). Pediatric-focused nonsurgeon nonproceduralists (2897 physicians) were significantly less likely to be associated with a CORS report than nonpediatric nonsurgeon nonproceduralists (15 391 physicians) (105 [3.6%] vs 927 [6.0%]; difference in percentages, −2.4 percentage points, 95% CI, −3.2 to −1.6 percentage points; *P* < .001). Pediatric-focused emergency medicine physicians, nonsurgeon proceduralists, and surgeons had no significant differences in reporting compared with nonpediatric-focused physicians.

**Conclusions and Relevance:**

In this cohort study, less than 10% of physicians ever received a coworker report with a concern about unprofessional behavior. Monitoring reports of unprofessional behaviors provides important opportunities for health care organizations to identify and intervene as needed to support team members.

## Key Points

**Question:**

Are there differences by specialty in the proportion of physicians who are identified in safety event reports submitted by coworkers describing unprofessional behaviors?

**Findings:**

In this cohort study of 35 120 physicians, 9.1% had at least 1 report from a coworker describing unprofessional behavior. Surgeons were most likely to receive a coworker report, and physicians with a pediatric focus were the least likely to receive a report of unprofessional behavior.

**Meaning:**

Understanding more about the distribution and patterns of unprofessional behaviors in health care that interfere with individual and team performance can support coworker well-being and the ability to deliver safe high-quality care.

## Teaser

This cohort study evaluates whether there are differences by physician specialty in the prevalence of reports of unprofessional behavior from coworkers.

## Introduction

High-functioning teams in health care, which include physicians, clinical staff, patients, and families, are essential to promote safe health outcomes. Most health care professionals consistently model professionalism toward coworkers, defined as clear communication; respect for patients, colleagues, and established safety practices; commitment to excellent technical care; and integrity.^1^ However, a small number of clinicians account for a disproportionate share of reports of unprofessional behaviors.^2,3,4^ Unprofessional behaviors threaten individual and team function^5,6^ and increase avoidable patient complications^7^; individuals who model unprofessional behaviors are associated with well-being concerns^8,9^ and increased malpractice claims.^10^

The Coworker Concern Obversation Reporting System (CORS) program is a national collaborative directed by the Vanderbilt Health Center for Patient and Professional Advocacy (CPPA) at Vanderbilt University Medical Center. A total of 193 participating hospitals and practice sites send electronic safety event reports describing concerns about unprofessional behaviors to CPPA, which uses qualitative coding^11^ to identify individual reports of unprofessional behavior as well as individual clinicians with repeated concerns.^2,4^ Individual reports and patterns of repeated concerns are used to identify clinicians who then receive feedback as a part of tiered interventions designed to promote self-regulation and to reduce reports of unprofessional behavior.^2^

The CORS program addresses behaviors by physicians, advanced practice clinicians, and nursing professionals. For the purposes of this study, we were interested in focusing on differences among physicians defined by their specialty because of the varying needs of teams that deliver care in different settings defined by physician specialty. Studies describing unprofessional behaviors among physicians toward coworkers have typically included all specialties and have used simulations or other models to identify the impact of unprofessional behavior.^2,3,5,6^ Limited data are available to understand the distribution and types of unprofessional behaviors among physicians by specialty and the proportion of physicians with repeated professionalism concerns. Understanding more about the distribution and patterns of repeated unprofessional behaviors can offer insight to leaders and organizations in supporting individual and team well-being and delivering safe care.

This study was designed to address the following study questions: what is the distribution of physicians by specialty who have received with reports of perceived unprofessional behaviors, measured through electronic safety reports in CORS? What are the types of concerns described in coworker reports about unprofessional behavior? Are there differences by specialty among physicians who develop apparent patterns of unprofessional behavior as described in CORS reports?

## Methods

### Study Design and Participants

This retrospective cohort study included credentialed physicians (ie, no residents or fellows) in the national CORS collaborative who had at least 1 day of active practice at a CORS site during the study period (January 1, 2018, to December 31, 2022). All physicians were eligible to be identified in a report. Physicians entered the cohort either on the first day of the study period or the physician’s first date of employment at an active CORS site, whichever came later. Physicians exited the cohort on the last day of the study period or the physician’s departure from the site. If a physician moved to another CORS site, only the first site’s data were used.

Research datasets were produced from existing data collected in support of the CORS process at each site. The dataset was deidentified without physician identifiers by a computer analyst at Vanderbilt University Medical Center who was not involved in the conduct of the research. Because the analysis was conducted on datasets that could not be linked to an individual, the Vanderbilt University Medical Center institutional review board determined that the study did not qualify as human participant research or require informed consent per CFR §46.102(e)(1). The study followed the Strengthening the Reporting of Observational Studies in Epidemiology (STROBE) reporting guideline for conducting cohort studies.^12^

### CORS Data

CORS data for each site are held at Vanderbilt under the terms of business associate agreements in place between each site and Vanderbilt University Medical Center. Each site provides names and practice specialties for credentialed physicians. At each site, reports of coworker concerns about a professional colleague’s behavior are entered into the health system’s electronic safety event reporting systems by any individual with access to the electronic safety reporting system.

Data are sent to Vanderbilt, and all patient, staff, and professionals’ identifiers are removed. Independent coders review CORS reports and reliably assign reports to various categories defined in previous studies.^2,11^ Coders assign unique concerns to categories, including clear and respectful communication, professional responsibility, competent medical care, and integrity.^11^ Coders are tested for reliability every 6 months and are found to have consistent coding 88% to 92% of the time. Reports with statements such as “Dr XX was about to start the bronchoscopy, but I reminded him we needed to do the time out. He mumbled, ‘You’re a bossy cow’” would be identified as disrespectful communication (eTable 1 in Supplement 1). “The patient was confused and had no idea where he was. I asked Dr YY if we should wait for the patient’s spouse to arrive. Dr YY said, ‘We’ll be here all night if we do that. If you won’t sign as a witness, I’ll get someone else who will’” includes statements that would be coded to professional responsibility. Reports about medical care might include reports such as “Dr XX removed the patient’s foley catheter at the end of the case without wearing gloves… had visible urine on his hands and did not wash them… proceeded to touch things in the operating room and left the room.” Reports about integrity would include reports such as, “Dr ZZ billed the visit as a level 5 visit, but I know they only spent 4 minutes with the patient.”

### Study Outcomes

The outcomes of interest were physicians’ total number and categories of CORS reports during the study period.^11^ Patterns were defined using a proprietary algorithm, which weights CORS reports based on recency and severity, with more recent and more severe reports contributing more than older and less severe reports.^2^ Physicians who are associated with CORS reports are supported by a tiered intervention model, including peer-delivered messages for single reports and awareness interventions for those with apparent patterns of reports, in which their status relative to local and national benchmarks is provided to them in an effort to guide self-regulation and improved performance.^2^

### Study Variables

Study variables included physician specialty identified from credentialing file categories, which were grouped as nonsurgeon nonproceduralists, emergency medicine physicians, nonsurgeon proceduralists, and surgeons, similar to previous studies (eTable 2 in Supplement 1).^13,14,15,16^ To assess whether previous findings showing that pediatric-focused clinicians generated fewer professionalism concerns reported by patients might also apply to coworker concerns,^16^ physicians whose credentialing files included a pediatric specialty or subspecialty were identified as pediatric focused. Site characteristics included the region of the country and whether the physician’s site of practice was academic or community (ie, regional health system or community-based multispecialty group).

### Statistical Analysis

We calculated the proportion of physicians in each specialty who received at least 1 report and who qualified for an awareness intervention based on having a pattern of repeated professionalism concerns. Proportions of physicians in each specialty having single reports and those qualifying for awareness interventions were reported with a 95% CI and compared using the Pearson χ^2^ test. In addition, the proportion of clinicians receiving at least 1 report of each type was reported by specialty. Logistic regression was used to calculate odds of any CORS report, adjusting for specialty, region, academic practice status, and pediatric specialty status. Adjusted odds ratios (ORs) with 95% CIs were reported by specialty and by pediatric focus. Two-sided *P* values of less than .05 were considered statistically significant. Sensitivity analyses restricted to physicians with at least 2 years of follow-up (99% of the original cohort) were not materially different than primary models, so analyses reported here include the full cohort. All statistical analyses were performed with R version 4.2.3 (R Project for Statistical Computing) from April 14 to September 8, 2023.

## Results

The cohort included 35 120 physicians, with 18 288 (52.1%) nonsurgeon nonproceduralists, 1876 (5.3%) emergency medicine physicians, 6743 (19.2%) nonsurgeon proceduralists, and 8213 (23.4%) surgeons (Table 1). The largest proportion of physicians practiced in the Midwest region of the United States, and the second largest proportion practiced in the Northeast region, with the exception of emergency medicine physicians, for whom the second largest proportion practiced in the West. The largest proportion of physicians practiced in academic settings, reflecting the distribution of CPPA sites.^7^ There were 4705 physicians (13.4%) who practiced in pediatric settings. Surgeons had the smallest proportion of physicians with a pediatric-focused practice (451 [5.5%]).

**Table 1.** Characteristics of the Cohort

Characteristic	Physicians, No. (%)					*P* value^a^
	Nonsurgeon nonproceduralist (n = 18 288)	Emergency medicine (n = 1876)	Nonsurgeon proceduralist (n = 6743)	Surgeon (n = 8213)	Total (N = 35 120)	
Region						
Midwest	7568 (41.4)	941 (50.2)	2570 (38.1)	3089 (37.6)	14 168 (40.3)	<.001
Northeast	4332 (23.7)	284 (15.1)	1749 (25.9)	2360 (28.7)	8725 (24.8)	
South	2342 (12.8)	247 (13.2)	1004 (14.9)	1027 (12.5)	4620 (13.2)	
West	4046 (22.1)	404 (21.5)	1420 (21.1)	1737 (21.1)	7607 (21.7)	
Practice type						
Academic	11 173 (61.1)	955 (50.9)	4498 (66.7)	5199 (63.3)	21 825 (62.1)	<.001
Community	7115 (38.9)	921 (49.1)	2245 (33.3)	3014 (36.7)	13 295 (37.9)	
Pediatric focus status						
Nonpediatric focus	15 391 (84.2)	1631 (86.9)	5631 (83.5)	7762 (94.5)	30 415 (86.6)	<.001
Pediatric focus	2897 (15.8)	245 (13.1)	1112 (16.5)	451 (5.5)	4705 (13.4)	

^a^ Pearson χ^2^ test.

**Figure 1.**
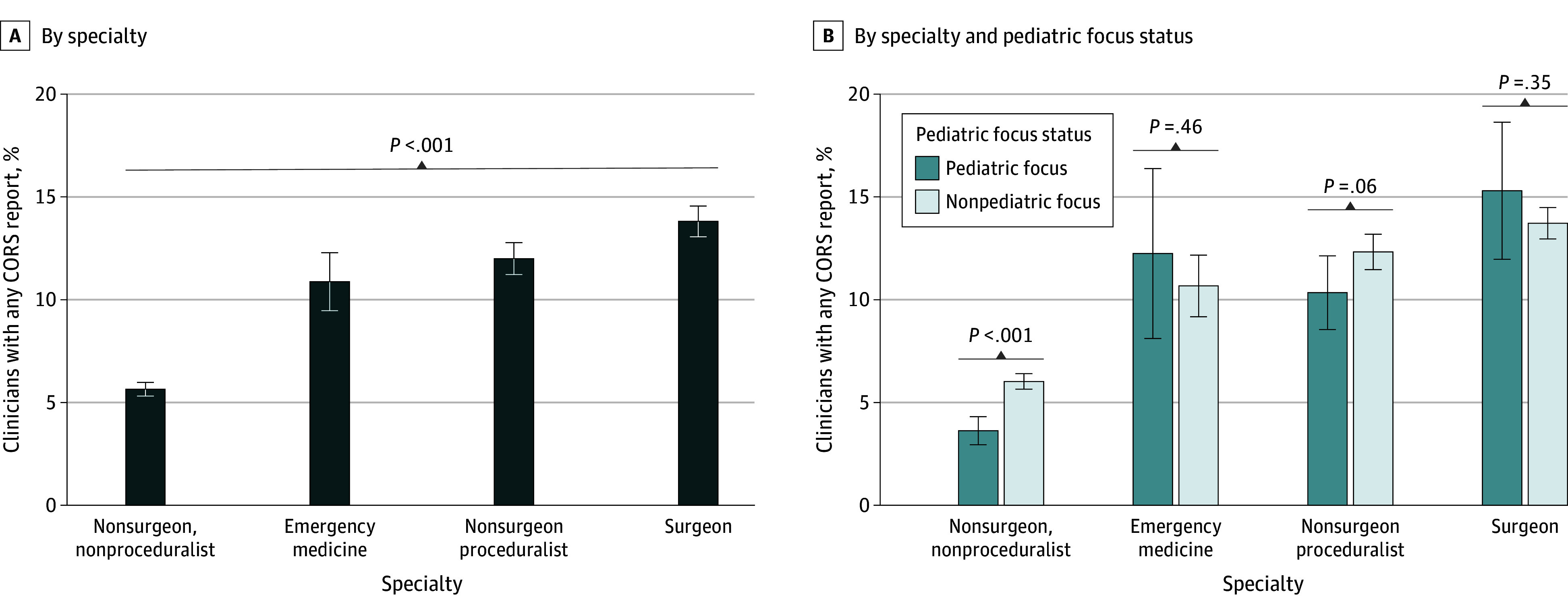
Proportion of Physicians by Specialty and Pediatric Focus Identified in 1 or More Coworker Concern Reports Error bars indicate 95% CIs for percentages. *P* value shows significance of test for difference in percentages between all groups using the Pearson χ^2^ test. CORS indicates Coworker Concern Observation Reporting System.

There were 3179 physicians (9.1%) who were associated with at least 1 CORS report. The proportion of individual physicians named in at least 1 CORS report differed by specialty (Figure 1). Nonsurgeon nonproceduralists had the lowest percentage of physicians associated with at least 1 report (1032 physicians [5.6%]), followed by emergency medicine (204 [10.9%]), nonsurgeon proceduralists (809 [12.0%]), and surgeons (1134 [13.8%]). Nonsurgeon nonproceduralists were significantly less likely to be named in a CORS report than the other specialties (1032 [5.6%] vs 2147 [12.8%] for other specialties combined; difference in percentages, −7.1 percentage points; 95% CI, −7.7 to −6.5 percentage points; *P* < .001). Overall, physicians who were pediatric focused (4705 physicians) were significantly less likely to have at least 1 CORS report than physicians who were not pediatric focused (30 415 physicians) (319 [6.8%] vs 2860 [9.4%]; difference in percentages,−2.6 percentage points; 95% CI, −3.4 to −1.8 percentage points; *P* < .001). Pediatric-focused nonsurgeon nonproceduralists (2897 physicians) were significantly less likely to be associated with a CORS report than nonpediatric-focused nonsurgeon nonproceduralists (15 391 physicians) (105 [3.6%] vs 927 [6.0%]; difference in percentages, −2.4 percentage points; 95% CI, −3.2 to −1.6 percentage points; *P* < .001) (Figure 1A). For other specialty focus areas, there was no statistically significant difference in the proportion of physicians associated with having at least 1 CORS report between those with a pediatric focus and those with a nonpediatric focus.

**Figure 2.**
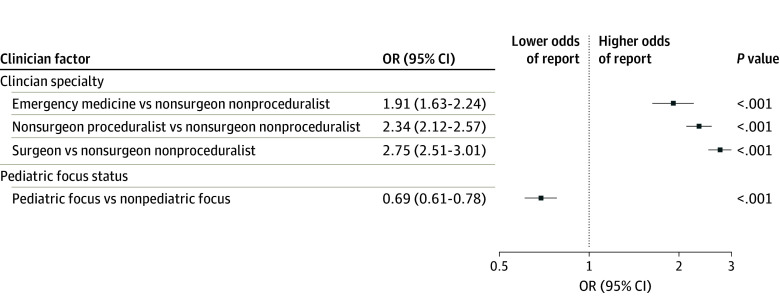
Multivariable Analysis of Odds of Receiving a Coworker Concern Report by Specialty and by Pediatric Focus Adjusted odds ratios (ORs) of at least 1 coworker concern report of any type, with 95% CI. Effect estimates derived using logistic regression adjusting for clinician specialty, pediatric focus status, region, and academic practice setting status.

In a multivariable logistic regression model controlling for physician and practice site characteristics (Figure 2), all specialty types had significantly higher odds of at least 1 coworker concern report when compared with nonsurgeon nonproceduralists, including emergency medicine physicians (adjusted OR, 1.91; 95% CI, 1.63-2.24), nonsurgeon proceduralists (adjusted OR, 2.34; 95% CI, 2.12-2.57), and surgeons (adjusted OR, 2.75; 95% CI, 2.51-3.01) (*P* < .001). Pediatric-focused physicians were significantly less likely to have a coworker concern report than those with a nonpediatric focus (adjusted OR, 0.69; 95% CI, 0.61-0.78; *P* < .001).

The most common types of CORS reports for all physician specialties involved clear and respectful communication, followed by professional responsibility (Table 2). The least common type of CORS report involved professional integrity. There was a statistically significant difference between specialties in the proportion having reports for each type of CORS report with nonsurgeon nonproceduralists demonstrating the lowest rate of reports of each type.

**Table 2.** Types of Coworker Concern Reports Received by Physician Specialty Group

Report type	Physicians, No. (%)					*P* value^a^
	Nonsurgeon nonproceduralist (n = 18 288)	Emergency medicine (n = 1876)	Nonsurgeon proceduralist (n = 6743)	Surgeon (n = 8213)	Total (N = 35 120)	
Any CORS report	1032 (5.6)	204 (10.9)	809 (12.0)	1134 (13.8)	3179 (9.1)	<.001
Communication	782 (4.3)	160 (8.5)	668 (9.9)	921 (11.2)	2531 (7.2)	<.001
Responsibility	549 (3.0)	89 (4.7)	392 (5.8)	529 (6.4)	1559 (4.4)	<.001
Medical care	150 (0.8)	49 (2.6)	186 (2.8)	241 (2.9)	626 (1.8)	<.001
Professional integrity	166 (0.9)	42 (2.2)	121 (1.8)	180 (2.2)	509 (1.4)	<.001

Abbreviation: CORS, Coworker Concern Observation Reporting System.

^a^ Pearson χ^2^ test.

**Figure 3.**
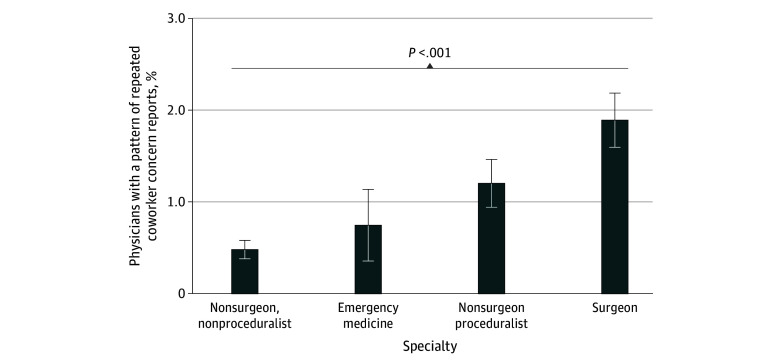
Proportion of Physicians With Repeated Coworker Concern Reports by Specialty Error bars represent 95% CIs for percentages. *P* value shows significance of test for difference in percentages between all groups using Pearson χ^2^ test.

There were 338 physicians (1.0%) who had repeated CORS reports that represented a pattern. The proportion of physicians who had a pattern of CORS reports ranged from 0.5% (88 of 18 288 nonsurgeon nonproceduralists) to 1.9% (155 of 8213 surgeons) (Figure 3). Emergency medicine physicians (14 of 1876 [0.7%]) and nonsurgeon proceduralists (81 of 6743 [1.2%]) had incrementally higher proportions of individuals with a pattern of repeated CORS reports than nonsurgeon nonproceduralists (*P* = .001).

## Discussion

In this cohort study of 35 120 physicians, 9.1% were associated with at least 1 report about unprofessional behavior by coworkers, meaning that most physicians never received any reports. The proportion receiving at least 1 CORS report ranged from 5.6% to 13.8% with observed differences by specialty focus. In a multivariate model, physicians who practiced in nonsurgical, nonprocedural–focused specialties were significantly less likely to have CORS reports than the other specialties. Physicians who had a pediatric focus were significantly less likely to be identified in a CORS report than physicians who did not have a pediatric focus. There was an even smaller proportion (<1%) of physicians with a pattern of repeated CORS reports. The proportion of individuals associated with a pattern in each specialty differed significantly, with surgeons being the group most likely to be associated with a pattern of repeated CORS reports.

Unprofessional behavior observed and described by patients and coworkers has been shown to result in increased risk for patient complications^7,17^ and malpractice claims.^17^ Physicians with repeated professionalism concerns are also likely to have a deleterious impact on culture and team performance.^5,6,17^ What are ways in which unprofessional behaviors might increase the risk for complications? Exposure to dismissive behaviors has been shown to reduce individual clinician and team performance, often mediated through reduced vigilance, help-seeking, and communication.^5,6^ Individuals and teams who are more focused on the unprofessional colleague may not pay as much attention to the task at hand or may not speak up if they have concerns about clinical care. In this study, concerns about unprofessional communication were the most common reports and concerns about professional integrity were the least common. The types and frequencies of unprofessional behavior types in this study parallel the distribution seen in previous studies.^3,7,11^

Surgeons were more likely to have at least 1 CORS report of unprofessional behavior and to have patterns of CORS reports than other specialties. Previous studies have shown higher risk among surgeons for being named in unsolicited patient complaints and malpractice claims.^10,18,19,20^ It is possible that surgeons practice in more stressful environments than the other specialties, resulting in interactions during high-stakes events that increased the likelihood of a coworker reporting a concern. In addition, surgeons serve on teams that require interdependence, potentially increasing the frustration for the surgeon or for other members of the team. It is also possible that personality characteristics of surgeons, nurses, and other clinicians who deliver care in perioperative settings differ from clinicians who provide care in other settings. These dynamics could affect both the frequency of unprofessional behaviors and the likelihood that an individual would report the behavior.

Nonsurgeon proceduralists are likely to practice in both procedural areas (ie, endoscopy suites) and inpatient and ambulatory settings, so individual team member’s perceptions of unprofessional behavior could be governed by the degree of complexity and stress in those environments. In addition, team-based care for nonsurgeon procedural specialties could introduce additional stresses and tension points that might affect team performance. Emergency medicine physicians were less likely than surgeons and nonsurgeon proceduralists to be associated with a coworker concern. The structure of teams in emergency medicine and the interaction of emergency medicine physicians with multiple professions and specialties introduces unique stresses on team function in that setting. In addition, care in emergency departments can be stressful and overwhelming at times. Interestingly, physicians who practiced with pediatric-focused privileges were significantly less likely to receive a CORS report even when controlling for specialty and practice setting. There may be a difference in physicians and nurses who choose to practice in pediatrics, possibly reflecting a different temperament or practice style. Overall, however, regardless of specialty, the majority of physicians in this cohort (91% overall) never received any reports of unprofessional behavior during the study period.

The findings of this study have implications for both individual physicians and health care leaders. Understanding more about how different team members in complex health care teams perceive behaviors provides insight into culture, psychological safety, and trust in a variety of settings. Previous studies have demonstrated that physicians with well-being concerns are more likely to be associated with reports of unprofessional behavior.^8,9,21^ Physicians who develop patterns of unprofessional behavior reports may need an assessment of their well-being. In a similar fashion, if entire groups of physicians begin to receive reports more than other groups, systems issues, culture, or leadership factors should be considered.^22^ It is worth noting that 86% to 90% of physicians by specialty did not have any CORS reports during the study period. Health care leaders can use these data to underscore the importance of professional accountability in creating optimal patient outcomes and individual and team function.^1,2,17^

### Limitations

This study has important limitations. It is possible that some physicians modeled unprofessional behaviors that went unreported because of individual’s fear of retaliation or lack of psychological safety.^23^ Other environmental variables related to the physician’s practice that are unmeasured here could contribute to either unprofessional behaviors or to differential reporting when behaviors occur. Reports were not investigated to determine whether the reporter’s perception was true or not. The assignment of physicians to specialty was based on credentialing files that may not reflect the physician’s actual practice. For example, some cardiologists might perform procedures, while others may not, which would bias the findings to the null, since nonproceduralists were identified in fewer reports as a group. It is possible that physicians identified with a pediatric focus may have a range of practice exposure in pediatric settings. For example, individuals who practice in a large center with highly specialized care (ie, a children’s hospital) may have a primary focus on pediatric care, while individuals in other settings may deliver care to children and adults and thus may share attributes more in common with nonpediatric clinicians. The bias of such misclassification of pediatric focus would bias the findings toward the null. Even though the study cohort included more than 35 000 physicians, it was not possible to do extensive analyses among specific specialties and subspecialties. In addition, study records did not include gender; in previous studies, women were less likely to receive CORS reports.^3,7^ The study included a greater proportion of academic than community-based centers, so the cohort may not be representative of the general population of physicians.

## Conclusions

In this study, less than 10% of physicians received a coworker report for unprofessional behavior during the study period. Physicians who practiced in pediatric settings were the least likely to receive a report about unprofessional behavior. Surgeons and proceduralists were more likely to receive a CORS report than nonsurgeon nonproceduralists and emergency medicine physicians. Because unprofessional behaviors are associated with patient complications, malpractice claims, and well-being concerns, monitoring concerning behavior and especially those physicians with repeated reports provides important opportunities for physicians and leaders to support professionalism, which increases the chance of health care organizations meeting their clinical, cultural, and other performance goals.

## Notes

Supplement 1. **eTable 1.** Examples of Coworker Observation Reporting System Reports by Type and Specialty **eTable 2.** Physician Specialty Grouping

Supplement 2.
Data Sharing Statement
