# Error in Results and Figure

**DOI:** 10.1001/jamanetworkopen.2024.31267

**Published:** 2024-08-07

In the Original Investigation titled “Physician Specialty Differences in Unprofessional Behaviors Observed and Reported by Coworkers,”^1^ published June 6, 2024, there were errors in the Results section and Figure 2. In the last paragraph of the Results section, the number of nonsurgeon nonproceduralists with a pattern of reports should have read 88 of 18 288. In Figure 2, the adjusted odds ratios for having at least 1 coworker concern compared with nonsurgeon nonproceduralists should have been 1.91 (95% CI, 1.63-2.24) for emergency medicine physicians, 2.34 (95% CI, 2.12-2.57) for nonsurgeon proceduralists, and 2.75 (95% CI, 2.51-3.01) for surgeons. The adjusted odds ratio comparing pediatric vs nonpediatric focus should have been 0.69 (95% CI, 0.61-0.78). This article has been corrected.^1^
