# Supplementary material for: Physician Specialty Differences in Unprofessional Behaviors Observed and Reported by Coworkers
Source: JAMA Netw Open. 2024 Jun 6;7(6):e2415331. doi: 10.1001/jamanetworkopen.2024.15331 (PMC11157352; doi:10.1001/jamanetworkopen.2024.15331)
Supplement: Supplement 1. — eTable 1. Examples of Coworker Observation Reporting System Reports by Type and Specialty eTable 2. Physician Specialty Grouping [file jamanetwopen-e2415331-s001.pdf]

# Supplemental Online Content

Cooper WO, Hickson GB, Dmochowski RR, et al. Physician specialty differences in unprofessional behaviors observed and reported by coworkers. *JAMA Netw Open.* 2024;7(6):e2415331. doi:10.1001/jamanetworkopen.2024.15331

**eTable 1.** Examples of Coworker Observation Reporting System Reports by Type and Specialty

**eTable 2.** Physician Specialty Grouping

This supplemental material has been provided by the authors to give readers additional information about their work.

© 2024 Cooper WO et al. *JAMA Network Open.*

**eTable 1. Examples of Coworker Observation Reporting System Reports by Type and Specialty**

| Coded Category | Non-surgeon, non-proceduralist | Emergency Medicine | Non-surgeon proceduralist | Surgeon |
|---|---|---|---|---|
| **Clear and Respectful Communication** | "Dr. ___ gave me a vague answer about dose and then refused to answer any further questions." | "Dr. ___ said, 'It took you an hour to get here for the consult. I hope you enjoyed your latte." | "Dr. ___ yelled at me in the holding room in front of patients and families." | "Dr. yanked the instrument out of my hand and said, 'Give me that and let's find someone who can do their job." |
| **Professional Responsibility** | "I told Dr. ___ we couldn't do verbal orders in clinic. Dr. said, 'Figure it out," and turned and walked away." | "I tried to explain to Dr. ___ that our quality review had found some opportunities for the last resuscitation they lead. Dr. __ said, 'Once you've done half as many resuscitations as I've done, we can talk.'" | "Dr. ____ always yells about getting the first case started on time. I paged Dr. ___ about getting consent for the first case and Dr. ____ never returned my call." | "I told Dr. ____ that the protocol called for changing gloves for this case. Dr. _____ said, 'The entire case is dirty. Changing gloves is not necessary.' Dr. ____ continued without changing gloves." |
| **Competent Medical Care** | "Dr. ___ seemed confused and did not seem to remember a patient he had just seen two days ago." | "Dr. ___ started suturing while the child was still screaming. We asked Dr. ___ to wait. Dr. ___kept going." | "Dr. ___ dropped the scope on the floor, picked it up and proceeded with the case." | "Dr. ___ told me I had to change the drain setting. I told Dr. __ we are not allowed to make those |

© 2024 Cooper WO et al. *JAMA Network Open.*

| | | | | |
|---|---|---|---|---|
| | | | | changes per policy. Dr. __ smirked and said, 'Just do it.'" |
| **Integrity** | "The patient waited for an hour and left before Dr. ___ saw them. Dr. ___ wrote a note that said that they saw the patient." | "Dr. ___ talked about a patient's private information in front of several patients on stretchers in the hallway." | "Dr. ___ billed the visit as a level 5 visit, but I know they only spent 4 minutes with the patient." | "Dr. ___ told the scheduler to overbook the morning clinic so that they could meet their bonus quota." |

**eTable 2. Physician Specialty Grouping**

| Non-Surgeon<br>Non-Procedural Specialties |
|---|
| |
| **Pediatrics** |
| General Pediatrics |
| Adolescent Medicine |
| **Pediatric Internal Medicine** |
| Nephrology-Pediatric |
| Allergy/Immunology-Pediatric |
| Dermatology-Pediatric |
| Endocrinology-Pediatric |
| Hematology/Oncology-Pediatric |
| Hospitalist-Pediatric |
| Infectious Disease-Pediatric |
| Internal Medicine-Pediatric |
| **Pediatric Other** |
| Neurology-Pediatric |
| Rheumatology-Pediatric |
| Pathology-Pediatric |
| Psychiatry-Pediatric |
| **Internal Medicine** |
| Dermatology |
| Endocrinology |
| Reproductive Endocrinology |
| Family Medicine |
| Medical Genetics |
| Geriatric Medicine |
| Hematology/Oncology |
| Hepatology |
| Hospitalist |
| Infectious Disease |
| Internal Medicine |
| Pharmacology |
| Nephrology |
| Pain |
| Palliative Medicine |
| Sleep |
| Allergy/Immunology |
| **Radiology** |
| Nuclear Medicine |
| Diagnostic Radiology |
| Radiology |
| Neuroradiology |

© 2024 Cooper WO et al. *JAMA Network Open.*

| |
|---|
| **Ortho** |
| Occupational Medicine |
| Podiatry |
| **Other** |
| Headache |
| Neurophysiology |
| None |
| Pathology |
| Psychiatry |
| Radiation Oncology |
| Physical Medicine/Rehabilitation |
| Rheumatology |
| Sickle Cell |
| Sports |
| Telemedicine |
| Transgender Medicine |
| |
| **Emergency Medicine Specialties** |
| |
| Emergency Medicine |
| Emergency Medicine-Pediatric |
| **Other** |
| Urgent Care |
| |
| **Non-Surgeon, Procedural Specialties** |
| |
| **Pediatric** |
| Neonatology |
| **Pediatric/Internal Medicine** |
| Pulmonary Disease -Pediatric |
| Critical Care-Pediatric |
| Cardiology – Pediatric |
| Gastroenterology-Pediatric |
| **Pediatric/Anesthesiology** |
| Anesthesiology-Pediatric |
| **Internal Medicine** |
| Gastroenterology |
| Critical Care |
| Cardiac Electrophysiology |
| Cardiology |
| Interventional Cardiology |
| Pulmonary Disease |
| **Radiology** |

© 2024 Cooper WO et al. *JAMA Network Open.*

| |
|---|
| Interventional Radiology |
| **Anesthesiology** |
| Anesthesiology |
| **Other** |
| Neurology |
| Wound Care |
| |
| **Surgeon Specialties** |
| |
| **CT Surgery** |
| Cardio-Thoracic Surgery |
| Cardio-Thoracic Surgery-Pediatric |
| Thoracic Surgery |
| **General Surgery** |
| Colorectal Surgery |
| General Surgery |
| General Surgery-Pediatric |
| Bariatric Surgery |
| Trauma and Surgical Critical Care |
| Surgical Oncology |
| Plastic Surgery |
| Reconstructive Plastic Surgery |
| **Transplant** |
| Transplant |
| Transplant (Liver) |
| Transplant (Renal) |
| **Vascular** |
| Vascular Surgery |
| **Ortho** |
| Foot and Ankle |
| General Orthopaedics |
| Hand |
| Hip and Knee |
| Joint |
| Orthopedic Surgery |
| Orthopedic Surgery-Pediatric |
| Orthopedics-Pediatric |

© 2024 Cooper WO et al. *JAMA Network Open.*

| |
|---|
| Shoulder |
| Shoulder and Elbow |
| Spine |
| **Head and Neck** |
| Dentistry |
| Dentistry-Pediatric |
| Oral Surgery |
| Otolaryngology |
| Otolaryngology-Pediatric |
| Otorhinolaryngology |
| Head and Neck |
| **Genitourinary** |
| Urogynecology |
| Urology |
| Urology-Pediatric |
| Female Pelvic Medicine and Reproductive Surgery |
| Gynecological Oncology |
| Maternal-Fetal Medicine |
| Obstetrics/Gynecology |
| **Other Surgical** |
| Neurosurgery |
| Neurosurgery-Pediatric |
| Ophthalmology |
| Ophthalmology-Pediatrics |

© 2024 Cooper WO et al. *JAMA Network Open.*
