# Supplementary material for: Physician Specialty Differences in Unprofessional Behaviors Observed and Reported by Coworkers
Source: JAMA Netw Open. 2024 Jun 6;7(6):e2415331. doi: 10.1001/jamanetworkopen.2024.15331 (PMC11157352; doi:10.1001/jamanetworkopen.2024.15331)
Supplement: Supplement 2. — Data Sharing Statement [file jamanetwopen-e2415331-s002.pdf]

Data Sharing Statement

Cooper. Physician Specialty Differences in Unprofessional Behaviors Observed and Reported by Coworkers. *JAMA Netw Open*. Published June 06, 2024.
doi:10.1001/jamanetworkopen.2024.15331

Data
Data available: No
